# Association between estimated glucose disposal rate and cardiovascular disease prevalence and mortality outcomes in metabolic dysfunction-associated steatotic liver disease: a comparative analysis of insulin resistance markers

**DOI:** 10.7189/jogh.15.04249

**Published:** 2025-08-04

**Authors:** Xiaoli Chen, Leilei Du, Jia Peng

**Affiliations:** 1 Department of Cardiovascular Medicine, Xiangya Hospital, Central South University, Changsha, Hunan, China; 2 National Clinical Research Center for Geriatric Disorders, Xiangya Hospital, Changsha, Hunan, China; 3 Laboratory of Cardiovascular Science, Beijing Clinical Research Institute, Beijing Friendship Hospital, Capital Medical University, Beijing, China

## Abstract

**Background:**

Metabolic dysfunction-associated steatotic liver disease (MASLD), primarily driven by insulin resistance (IR), is emerging as a significant public health concern. While the estimated glucose disposal rate (eGDR), a novel marker of IR, could be useful in predicting adverse outcomes in diabetes, its role in MASLD remains unclear.

**Methods:**

We used data from the National Health and Nutrition Examination Survey (NHANES) collected from 1999 to 2018, and combined cross-sectional and cohort study designs to explore the associations between eGDR, cardiovascular disease (CVD) outcomes in US adults with MASLD. Here, MASLD was defined using the fatty liver index or hepatic steatosis index along with cardiometabolic risk factors. We applied survey-weighted logistic regression to evaluate CVD prevalence and used Cox proportional hazards models to assess mortality risk. We analysed nonlinear associations between eGDR, CVD, and mortality outcomes using restricted cubic splines. Lastly, we compared the predictive performance of eGDR with traditional IR markers, including the triglyceride-glucose (TyG) index and HOMA-IR, *via* C-statistics.

**Results:**

Logistic regression and Cox proportional hazards models showed that lower eGDR levels were consistently associated with increased risks of CVD prevalence (*P* < 0.001) and mortality (*P* < 0.001), even after adjusting for potential confoundersin both MASLD models. eGDR also outperformed TyG and HOMA-IR in predicting all-cause and CVD mortality (*P* < 0.001), underscoring its superior prognostic value in MASLD populations (*P* < 0.001). Moreover, incorporating eGDR into the baseline model significantly enhanced predictive accuracy and reclassification (*P* < 0.001), further validating its potential to improve risk prediction.

**Conclusions:**

Lower eGDR levels are associated with higher risks of CVD and mortality in MASLD. The eGDR outperforms traditional IR markers in predicting all-cause mortality and improves risk prediction models, highlighting its potential usefulness for clinical risk stratification in MASLD.

Through a recent Delphi consensus process, experts introduced the term ‘steatotic liver disease’ as a replacement for the broader term ‘fatty liver disease’, while ‘metabolic dysfunction-associated steatotic liver disease’ (MASLD) has been adopted as the updated terminology for ‘nonalcoholic fatty liver disease’ to reflect its metabolic underpinnings better [1]. While non-alcoholic fatty liver disease was defined by hepatic steatosis in the absence of significant alcohol consumption or other liver diseases, MASLD requires evidence of hepatic steatosis in addition to at least one cardiometabolic risk factor, thereby shifting from a diagnosis of exclusion to one that highlights the central role of metabolic dysfunction [1]. According to the National Health and Nutrition Examination Survey (NHANES) database, the prevalence of MASLD in the US adult population is estimated at 32.45% [2,3]. This substantial prevalence, coupled with the direct medical costs that have been estimated at USD 103 billion annually in the USA alone [4], underscores the significant public health burden posed by MASLD.

Among the major contributors to MASLD pathogenesis, insulin resistance (IR) plays a pivotal role in the development and progression of MASLD itself, linking it to an increased risk of cardiovascular disease (CVD), diabetes mellitus (DM), and early mortality [5–9]. Given its clinical significance, an accurate assessment of IR is essential for risk stratification. Due to its high accuracy and reliability, the hyperinsulinemic-euglycemic clamp technique is considered the benchmark for evaluating insulin sensitivity [10]. However, its invasiveness, time-consuming nature, and operational complexity limit its application mainly to research settings [11]. Although more accessible markers such as the homeostasis model assessment of IR (HOMA-IR) [12] and the triglyceride-glucose (TyG) index [13] are widely used in clinical settings, they primarily reflect fasting-state metabolic activity and fall short in capturing the systemic and multifactorial nature of IR, thereby limiting their prognostic utility across varied populations [11,12,14,15]. In contrast, the estimated glucose disposal rate (eGDR) has emerged as a more integrative and comprehensive surrogate for IR. Unlike HOMA-IR and TyG, eGDR incorporates both metabolic (glycated hemoglobin A1c (HbA1c), waist circumference (WC)) and hemodynamic (hypertension parameters), allowing it to reflect a more systemic IR phenotype [15], which may be particularly relevant in the pathophysiology and clinical assessment of MASLD. Although eGDR was originally developed and validated in individuals with type 1 diabetes mellitus [16], subsequent studies have demonstrated its robust inverse association with the risks of stroke, coronary artery disease, and all-cause mortality across diverse populations, irrespective of diabetes status [15,17–20]. Furthermore, eGDR correlates with cardiovascular outcomes in individuals with metabolic syndrome [21], a condition marked by significant IR and metabolic heterogeneity that closely resembles the clinical features of MASLD. 

Epidemiological evidence indicates that individuals with MASLD face a substantially elevated risk of cardiovascular complications [7,22], with CVD consistently reported as the leading cause of death in this population [6,23]. A systematic review and meta-analysis of population-based studies reported all-cause and cardiac-specific mortality rates of 12.6 and 4.2 per 1000 person-years, respectively, among individuals with MASLD [24]. However, the specific association between eGDR and both CVD prevalence and mortality in MASLD populations remains poorly characterised. Given the established role of IR in the pathogenesis of MASLD and its association with adverse outcomes, further exploration of this link is warranted.

We aimed to explore the association between eGDR and CVD prevalence, as well as to evaluate its relationship with all-cause and CVD mortality in individuals with MASLD. Additionally, we sought to compare the predictive performance of eGDR with other commonly used IR markers, such as the TyG index and HOMA-IR, for mortality outcomes.

## METHODS

### Data source and participant selection

We retrieved data on 101 316 particiants from the NHANES database covering the period from 1999 to 2018. The NHANES employs a stratified, multistage probability sampling method to collect health data from a nationally representative sample of the US non-institutionalised population [25]. After applying our exclusion criteria, 6377 participants remained for analysis. Specifically, we excludedindividuals younger than 20 years (n = 46 235) or pregnant (n = 1541); those with insufficient data to calculate eGDR (n = 8782); individuals without a diagnosis of MASLD (n = 36 782); those without follow-up records (n = 6); and individuals missing covariate data or sample weights (n = 1593) (Figure S1 in the **Online Supplementary Document**).

### Diagnosis of MASLD

Given the lack of ultrasonographic assessment of hepatic steatosis in most NHANES interview cycles, we employed the fatty liver index (FLI), a validated surrogate for SLD [26,27], calculating it as follows [26]:



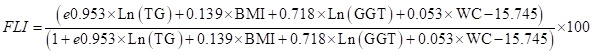



According to previous studies, we used an FLI cut-off value of 60 to classify participants with FLI >60 as having hepatic steatosis [1,28,29]. MASLD was characterised by the presence of SLD accompanied by at least one cardiometabolic risk factor, excluding cases of viral hepatitis (hepatitis B or hepatitis C) and excessive alcohol consumption (≥20 g/d for women and ≥30 g/d for men). Cardiometabolic risk factors comprise the following [1]: body mass index (BMI) ≥25 kg/m^2^ or WC ≥80 cm for females and ≥94 cm for males; FBG ≥100 mg/dL, two-hour post-load glucose ≥140 mg/dL, HbA1c ≥5.7%, or a diagnosis of type 2 diabetes mellitus; blood pressure ≥130/85 mm Hg; triglyceride levels ≥150 mg/dL; and high-density lipoprotein cholesterol <40 mg/dL for males or <50 mg/dL for females.

### Exposure and outcome variables

We derived eGDR using a formula reported in previous studies [18,30,31]:

eGDR = 21.158 − (0.09 × WC (cm)) − (3.407 × hypertension status (1 = yes, 0 = no)) − (0.551 × HbA1c in %).

We stratified participants into quartiles (Q1, Q2, Q3, Q4) according to their eGDR levels. The main outcomes we assessed were CVD prevalence, all-cause mortality, and CVD mortality. The CVD diagnoses were based on self-reported physician-confirmed diagnoses during personal interviews. Participants with a history of congestive heart failure, coronary heart disease, angina, myocardial infarction, or stroke were classified as having CVD [32]. We retrieved mortality data and follow-up durations from the NHANES linked mortality file, updated until 31 December 2019. We probabilistically matched these records to the National Death Index through an algorithm provided by the National Center for Health Statistics, with causes of death classified according to the International Classification of Disease, 10th revision.

### Clinical characteristics and covariates

We retrieved information on participants’ sociodemographic characteristics and living habits, comprising age, sex (female or male), race (Black, White, Mexican American, or other), educational attainment (under high school, high school or equivalent, and above high school), family income-to-poverty ratio (PIR) (<1.3, 1.3-3.5, ≥3.5), marital status (married or cohabiting vs other), smoking behaviour (never, former, or current) [33], and drinking status (yes or no). Physical and laboratory measurements included WC, BMI, energy intake, total cholesterol, triglycerides, HDL, HbA1c, FBG, uric acid, blood urea nitrogen, aspartate transaminase, alanine transaminase, serum creatinine and estimated glomerular filtration rate (eGFR).

### Statistical analysis

We followed NHANES analytic guidelines in conducting our statistical analyses, utilising sample weighting to account for the complex multi-stage survey design and to ensure national representation of US MASLD populations [34]. We addressed missing data using a complete-case approach, where we excluded individuals with missing values from the analysis. Given the NHANES weighting methodology, this approach minimised potential bias [34]. We used the Kolmogorov-Smirnov test to assess the normality of continuous variables, which we then presented as medians with interquartile ranges and compared across groups using the Kruskal-Wallis test for group comparisons. We described categorical as counts with corresponding weighted percentages and compared them between groups them using chi-squared tests.

We further used weighted univariable and multivariable logistic regression analyses to examine the link between eGDR and CVD in MASLD patients, and Cox proportional hazards models to investigate its relationship to all-cause and CVD-related mortality. We employed Kaplan-Meier curves to describe survival patterns and censored data for MASLD participants stratified by eGDR quartiles, with group differences evaluated through log-rank tests. We conducted restricted cubic spline analyses to evaluate the dose-effect associations between eGDR and CVD prevalence and mortality. Additionally, we performed subgroup analyses to examine associations across various demographic and clinical characteristics, including age, sex, race, PIR, education level, smoking status, alcohol use, obesity, and diabetes status.

Lastly, we assessed the predictive accuracy of CVD outcomes in MASLD participants using Harrell’s C-index [35], comparing its discriminatory ability against traditional IR indices such as the TyG index and HOMA-IR. We calculated the net reclassification improvement to evaluate the added predictive value of eGDR when incorporated into the baseline model.

To ensure the robustness of our findings, we performed sensitivity analyses using an alternative diagnostic criterion for MASLD – specifically, the hepatic steatosis index (HSI) of >36 as an indicator of SLD. This approach further validated the relationship between eGDR and CVD outcomes in MASLD individuals.

We used *R*, version 4.4.1 (R Core Team, Vienna, Austria) to conduct the statistical analyses. We applied Bonferroni correction to control for multiple testing based on a two-sided *P* = 0.05, resulting in an adjusted significance threshold of *P* = 0.017 (0.05/3).

## RESULTS

### Characteristics of the MASLD study cohort

We analysed data on 6377 participants (weighted to represent 62 579 003 individuals) diagnosed with MASLD. The cohort had a median age of 50 years, comprising 45.3% females and 54.7% males. Compared to the lowest quartile, the higher eGDR quartiles comprised more younger participants, Mexican Americans, drinkers, and never smokers, and was generally characterised by a lower prevalence of obesity and CVD history, elevated levels of total cholesterol and HDL, and reduced levels of WC, HbA1c, FBG, UA, and serum creatinine (**Table 1**).

**Table 1:** Baseline characteristics of participants with MASLD stratified by the eGDR*

	Total (n = 6377)	<4.14 (n = 1596)	4.14-5.49 (n = 1593)	5.49-8.11 (n = 1595)	>8.11 (n = 1593)	*P-*value
**Age, years, MD (IQR)**	50 (37–61)	56 (45–66)	56 (45–66)	47 (35–59)	42 (32–52)	<0.001
**Sex**						<0.001
Female	2971 (45.3)	731 (44.1)	793 (50.1)	793 (47.7)	654 (39.7)	
Male	3406 (54.7)	865 (55.9)	800 (49.9)	802 (52.3)	939 (60.3)	
**Race**						<0.001
Black	1165 (10.0)	418 (14.5)	316 (10.9)	241 (8.7)	190 (6.8)	
White	3024 (71.1)	774 (72.0)	802 (74.6)	782 (71.9)	666 (66.6)	
Mexican American	1347 (9.3)	226 (5.6)	292 (6.7)	349 (9.4)	480 (14.2)	
Other	841 (9.7)	178 (7.9)	183 (7.8)	223 (10.1)	257 (12.3)	
**Education levels**						0.027
Under high school	820 (6.3)	196 (6.8)	230 (6.8)	172 (4.9)	222 (6.9)	
High school or equivalent	2553 (38.3)	644 (37.9)	667 (41.6)	635 (38.2)	607 (35.9)	
Above high school	3004 (55.4)	756 (55.2)	696 (51.6)	788 (56.9)	764 (57.2)	
**Family income poverty ratio**						0.019
<1.3	1992 (21.5)	509 (21.8)	503 (20.4)	481 (21.3)	499 (22.5)	
1.3–3.5	2526 (38.3)	679 (41.5)	609 (37.1)	643 (40.3)	595 (35.0)	
≥3.5	1859 (40.1)	408 (36.7)	481 (42.5)	471 (38.5)	499 (42.5)	
**Marital status**						0.3
Married or living with a partner	4109 (67.8)	971 (65.2)	1018 (67.6)	1035 (68.5)	1085 (69.5)	
Others	2268 (32.2)	625 (34.8)	575 (32.4)	560 (31.5)	508 (30.5)	
**Smoking status**						<0.001
Never smoker	3313 (52.5)	770 (49.6)	790 (50.1)	863 (52.8)	890 (56.7)	
Former smoker	1932 (29.7)	578 (35.4)	552 (34.4)	432 (27.7)	370 (22.9)	
Current smoker	1132 (17.8)	248 (15.0)	251 (15.5)	300 (19.5)	333 (20.4)	
**Drinking status**						<0.001
Yes	4328 (71.9)	1008 (66.5)	1062 (70.6)	1082 (72.1)	1176 (77.2)	
No	2049 (28.1)	588 (33.5)	531 (29.4)	513 (27.9)	417 (22.8)	
**Energy intake in kcal, MD (IQR)**	2042.0 (1492.0–2688.0)	2013.0 (1482.3–2680.0)	1945.0 (1452.6–2633.0)	2042.0 (1481.7–2677.0)	2113.8 (1584.0–2730.0)	0.002
**BMI, kg/m^2^, MD (IQR)**	33.1 (30.1–37.5)	38.6 (34.5–43.3)	32.4 (30.2–35.2)	34.1 (30.5–37.9)	30.5 (28.6–32.9)	<0.001
**BMI category**						<0.001
Normal weight (<25)	81 (1.4)	2 (0.0)	12 (0.7)	23 (1.3)	44 (3.2)	
Overweight (25–30)	1528 (22.9)	83 (4.1)	403 (21.8)	388 (21.7)	654 (40.3)	
Obesity (≥30)	4768 (75.7)	1511 (95.8)	1178 (77.5)	1184 (77.0)	895 (56.6)	
**WC, cm, MD (IQR)**	110.7 (103.9–119.8)	124.4 (118.5–132.7)	109.5 (106.2–113.6)	114.9 (101.8–121.1)	103.7 (99.5–107.6)	<0.001
**TC, mg/dL, MD (IQR)**	197.0 (171.0–225.0)	186.0 (160.0–213.0)	196.0 (170.0–226.0)	200.0 (173.0–227.0)	205.0 (179.0–232.0)	<0.001
**TG, mg/dL, MD (IQR)**	146.0 (104.0–205.0)	141.0 (101.0–204.0)	146.0 (107.0–202.0)	141.0 (101.0–203.0)	153.0 (109.0–210.0)	0.015
**HDL, mg/dL, MD (IQR)**	45.0 (38.0–53.0)	44.0 (37.0–51.0)	45.0 (39.0–54.0)	45.0 (38.0–53.0)	44.0 (38.0–52.0)	0.001
**HbA1c, %, MD (IQR)**	5.60 (5.30–6.00)	6.10 (5.60–7.20)	5.60 (5.40–6.00)	5.50 (5.30–5.80)	5.30 (5.10–5.60)	<0.001
**Fast glucose, mg/dL, MD (IQR)**	103.0 (96.0–115.0)	117.0 (103.0–152.3)	105.0 (97.0–117.0)	102.0 (95.4–111.0)	99.0 (93.0–105.0)	<0.001
**ALT, U/L, MD (IQR)**	25.0 (19.0–34.0)	25.0 (18.0–33.0)	24.0 (18.0–32.0)	25.0 (19.0–34.0)	26.0 (20.0–35.0)	0.004
**AST, U/L, MD (IQR)**	23.0 (19.0–28.0)	23.0 (19.0–28.0)	23.0 (19.0–28.0)	23.0 (19.0–28.0)	23.0 (19.0–28.0)	0.6
**BUN, mg/dL, MD (IQR)**	13.00 (11.00–16.00)	14.00 (11.00–17.00)	14.00 (11.00–17.00)	13.00 (10.00–16.00)	13.00 (10.00–15.00)	<0.001
**UA, mg/dL, MD (IQR)**	5.90 (5.10–6.80)	6.20 (5.30–7.20)	5.90 (5.10–6.90)	5.90 (5.00–6.80)	5.80 (5.00–6.60)	<0.001
**Serum creatinine, mg/dl, MD (IQR)**	0.87 (0.73–1.00)	0.90 (0.74–1.03)	0.87 (0.72–1.00)	0.85 (0.71–1.00)	0.87 (0.74–1.00)	<0.001
**eGFR, ml/min/1.73m^2^, MD (IQR)**	97.1 (81.7–110.0)	93.2 (74.0–106.3)	91.7 (76.9–105.1)	99.1 (85.0–111.9)	102.9 (89.4–114.4)	<0.001
**eGDR, MD (IQR)**	5.81 (4.30–8.27)	3.08 (2.16–3.66)	4.79 (4.46–5.11)	6.94 (6.02–7.66)	8.83 (8.48–9.25)	<0.001
**CVD**	921 (11.7)	394 (22.1)	277 (13.8)	180 (9.5)	70 (3.6)	<0.001
**All-cause mortality**	1045 (12.3)	367 (18.2)	328 (16.1)	232 (10.7)	118 (6.0)	<0.001
**CVD mortality**	336 (3.7)	130 (6.6)	108 (4.8)	70 (2.9)	28 (1.2)	<0.001

ALT – alanine aminotransferase, AST – aspartate aminotransferase, BMI – body mass index, BUN – blood urea nitrogen, CVD – cardiovascular disease, eGFR – estimated glomerular filtration rate, HbA1c – glycated hemoglobin A1c, HDL – high-density lipoprotein cholesterol, PIR – family income-to-poverty ratio, TC – total cholesterol, TG – triglyceride, UA – uric acid, WC – waist circumference

*Data are presented as n (weighted %) unless specified otherwise.

### Association between eGDR and CVD in participants with MASLD

The prevalence of cardiovascular disease (CVD) decreased progressively across eGDR quartiles, with frequencies of 394 (22.1%), 277 (13.8%), 180 (9.5%), and 70 (3.6%) for Q1, Q2, Q3, and Q4, respectively. Multivariable-adjusted logistic regression analysis showed that, compared to the lowest eGDR quartile (Q1), participants in higher quartiles had a substantially lower prevalence of CVD (Q2: odds ratio (OR) = 0.93; 95% confidence interval (CI) = 0.90–0.96, *P* < 0.001; Q3: OR = 0.94; 95% CI = 0.91–0.96, *P* < 0.001; Q4: OR = 0.91; 95% CI = 0.89–0.94, *P* < 0.001) (**Table 2**). Restricted cubic spline analysis revealed a nonlinear association between eGDR and CVD (*P*-value for nonlinearity <0.001) (Figure S2 in the **Online Supplementary Document**). We identified a threshold effect at an eGDR value of 5.49, prompting further investigation with a two-piecewise logistic regression model. Below this threshold, each one-unit increase in eGDR was associated with a significantly reduced risk of CVD (OR = 0.98; 95% CI = 0.97–0.99, *P* < 0.001). However, when eGDR was ≥5.49, the association was no longer statistically significant (OR = 0.99; 95% CI = 0.99–1.00, *P* = 0.143), suggesting a potential saturation effect (Table S1 in the **Online Supplementary Document**).

**Table 2:** Associations between eGDR and prevalence of CVD among individuals with MASLD*

	Model 1		Model 2		Model 3	
**CVD**	**OR (95% CI)**	***P*-value**	**OR (95% CI)**	***P*-value**	**OR (95% CI)**	***P*-value**
**eGDR continuous**						
Per SD increase	0.93 (0.93, 0.94)	<0.001	0.96 (0.95, 0.97)	<0.001	0.97 (0.96, 0.98)	<0.001
**eGDR quartiles**						
Q1	ref		ref		ref	
Q2	0.92 (0.89, 0.95)	<0.001	0.93 (0.90, 0.96)	<0.001	0.93 (0.90, 0.96)	<0.001
Q3	0.88 (0.86, 0.91)	<0.001	0.93 (0.90, 0.96)	<0.001	0.94 (0.91, 0.96)	<0.001
Q4	0.83 (0.81, 0.86)	<0.001	0.90 (0.88, 0.93)	<0.001	0.91 (0.89, 0.94)	<0.001
*P*-value for trend		<0.001		<0.001		<0.001

CI – confidence interval, CVD – cardiovascular disease, eGDR – estimated glucose disposal rate, OR – odds ratio, Q – quartile, ref – reference, SD – standard deviation

*Model 1: no covariates were adjusted. Model 2: adjusted for age, gender, race, and family income poverty ratio. Model 3: adjusted for age, gender, race, family income poverty ratio, education level, marital status, smoking status, drinking status, energy intake, TC, ALT, AST, and eGFR.

**Figure 1:**
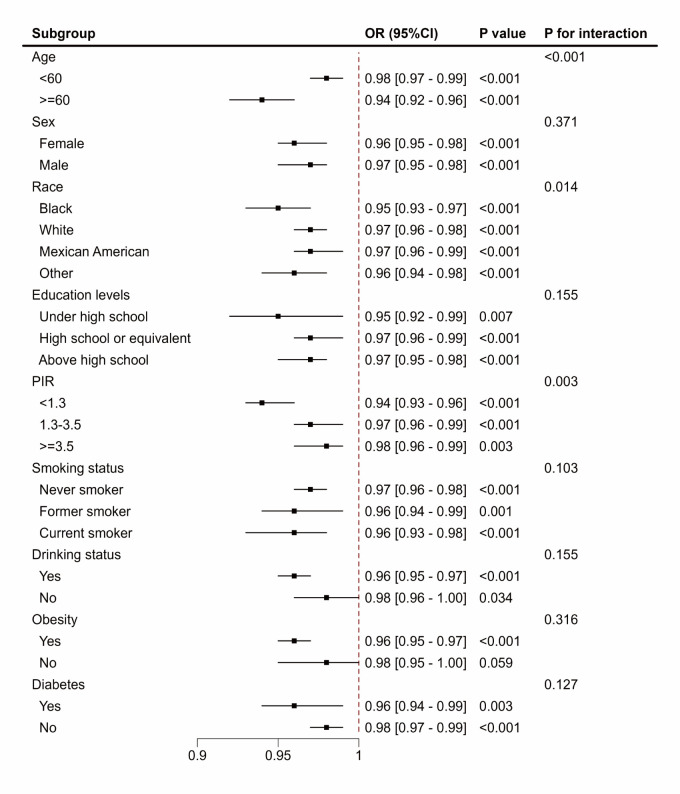
Subgroup analysis of the association between eGDR and CVD. Adjusted for age, gender, race, family income poverty ratio, education level, marital status, smoking status, drinking status, energy intake, TC, ALT, AST, and eGFR. CI – confidence interval, OR – odds ratio.

Subgroup analyses demonstrated consistent associations between eGDR and CVD across most demographic and behavioural factors, including age, sex, race, education level, PIR, smoking, alcohol use, obesity, and diabetes status. We observed no significant association in individuals without obesity. The interaction analyses showed significant effect modification by age, race, and PIR, highlighting that the relationship between eGDR and CVD varied according to these factors (**Figure 1**).

### Association of eGDR with all-cause and CVD mortality in MASLD populations

**Figure 2:**
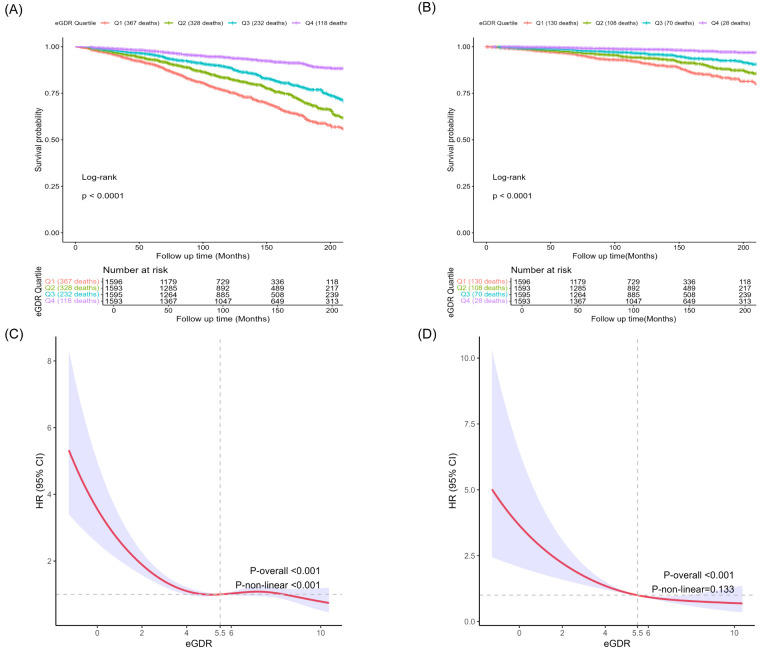
Kaplan-Meier survival analysis of eGDR quartiles with all-cause mortality (**Panel A**) and CVD mortality (**Panel B**) in the MASLD population. Restricted cubic spline analysis of the association between eGDR and all-cause (**Panel C**) and CVD (**Panel D**) mortality. Red lines represent references for ORs, and blue areas represent 95% CIs. The model was adjusted for age, gender, race, family income poverty ratio, education level, marital status, smoking status, drinking status, energy intake, TC, ALT, AST, eGFR, and CVD.

**Figure 3:**
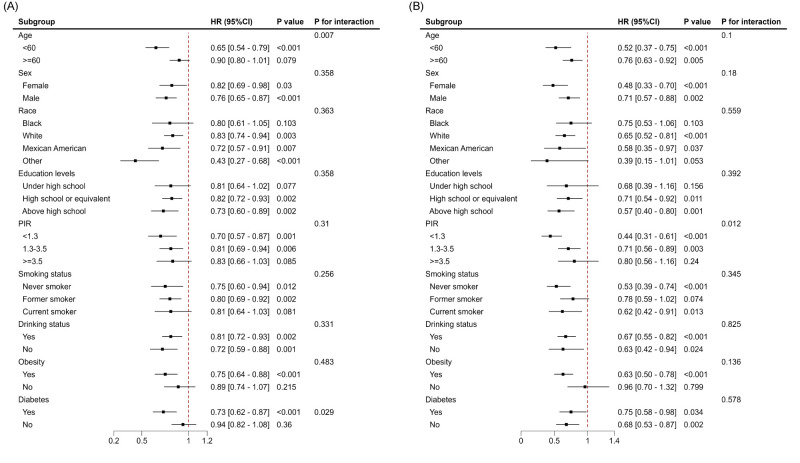
Subgroup analysis of the association between eGDR and all-cause (**Panel A**) and CVD mortality (**Panel B**) among individuals with MASLD. Adjusted for age, gender, race, family income poverty ratio, education level, marital status, smoking status, drinking status, energy intake, TC, ALT, AST, eGFR, and CVD. CI – confidence interval, HR – hazard ratio.

During a mean follow-up period of 9.74 years, 1045 deaths (12.3%) occurred among individuals with MASLD, including 336 deaths (3.7%) due to CVD. The results of the Kaplan-Meier curves indicated that MASLD participants in higher eGDR quartiles had significantly reduced all-cause mortality (*P* < 0.001) and CVD mortality (*P* < 0.001) compared to those in lower quartiles, as shown by the log-rank test (**Figure 2**, Panels A and B). The weighted Cox regression analysis showed that MASLD participants in the highest eGDR quartile had a markedly reduced risk of all-cause mortality (hazard ratio (HR) = 0.66; 95% CI = 0.55–0.79, *P* < 0.001) and CVD mortality (HR = 0.38; 95% CI = 0.22–0.64, *P* < 0.001) compared to those in the lowest quartile, after adjusting for covariates (**Table 3**). Restricted cubic spline analysis indicated a nonlinear association between eGDR and all-cause mortality, while the relationship between eGDR and CVD mortality was linear (**Figure 2**, Panels C and D). Given the nonlinear association with all-cause mortality, further analysis using a two-piecewise Cox model identified a threshold at eGDR of 5.50, below which each one-unit increase in eGDR was associated with an 18% reduction in all-cause mortality risk (HR = 0.82, 95% CI = 0.75–0.89, *P* < 0.001) and above which the association plateaued (HR = 1.02, 95% CI = 0.95–1.09; *P* = 0.650) (Table S2 in the **Online Supplementary Document**), suggesting a saturation effect. Subgroup analyses identified significant interactions between age and diabetes status with all-cause mortality and between PIR (*P* = 0.029) and CVD mortality (*P* = 0.012) (**Figure 3**). Specifically, the association between eGDR and all-cause mortality was stronger in individuals aged <60 years and those with diabetes. In contrast, for CVD mortality, a more pronounced effect was observed in participants with a PIR<1.3.

**Table 3:** Associations between eGDR and risk of all-cause and CVD mortality in the MASLD population*

	Model 1		Model 2		Model 3	
	**HR (95% CI)**	***P*-value**	**HR (95% CI)**	***P*-value**	**HR (95% CI)**	***P*-value**
**All-cause mortality**						
eGDR continuous						
*Per SD increase*	0.59 (0.55, 0.64)	<0.001	0.78 (0.70, 0.86)	<0.001	0.79 (0.71, 0.89)	<0.001
eGDR quartiles						
*Q1*	ref		ref		ref	
*Q2*	0.73 (0.60, 0.88)	<0.001	0.72 (0.60, 0.86)	<0.001	0.74 (0.61, 0.91)	0.003
*Q3*	0.49 (0.39, 0.61)	<0.001	0.72 (0.58, 0.88)	0.001	0.75 (0.60, 0.94)	0.011
*Q4*	0.23 (0.18, 0.31)	<0.001	0.58 (0.44, 0.76)	<0.001	0.62 (0.47, 0.81)	<0.001
*P*-value for trend		<0.001		<0.001		<0.001
**CVD mortality**						
eGDR continuous						
*Per SD increase*	0.50 (0.44, 0.57)	<0.001	0.63 (0.52, 0.77)	<0.001	0.66 (0.55, 0.79)	<0.001
eGDR quartiles						
*Q1*	ref		ref		ref	
*Q2*	0.59 (0.42, 0.82)	0.002	0.58 (0.42, 0.79)	<0.001	0.59 (0.43, 0.82)	0.002
*Q3*	0.36 (0.23, 0.55)	<0.001	0.55 (0.37, 0.83)	0.004	0.58 (0.38, 0.88)	0.011
*Q4*	0.13 (0.08, 0.21)	<0.001	0.35 (0.21, 0.58)	<0.001	0.38 (0.22, 0.64)	<0.001
*P*-value for trend		<0.001		<0.001		<0.001

CI – confidence interval, CVD – cardiovascular disease, eGDR – estimated glucose disposal rate, HR – hazard ratio, Q – quartile, ref – reference, SD – standard deviation

*Model 1: no covariates were adjusted. Model 2: adjusted for age, gender, race, and family income poverty ratio. Model 3: adjusted for age, gender, race, family income poverty ratio, education level, marital status, smoking status, drinking status, energy intake, TC, ALT, AST, eGFR, and CVD.

### Incremental predictive value of eGDR for all-cause and CVD mortality

The results of Harrell’s C-index, adjusted for the Bonferroni correction, showed that eGDR had a higher C-statistic compared to the individual IR indices (TyG index and HOMA-IR), significantly enhancing the predictive accuracy for both all-cause mortality (*P* < 0.001) and CVD mortality (*P* < 0.001) (Table S3 in the **Online Supplementary Document**). Additionally, when eGDR was added to the baseline model, we observed a significant improvement in predictive accuracy and reclassification (*P* < 0.001), further supporting its value in enhancing predictive performance.

### Sensitive analyses

Sensitivity analyses demonstrated that the association between eGDR and CVD prevalence and mortality remained consistent when MASLD was defined using the HSI, a validated surrogate marker for hepatic steatosis. Here, HSI was calculated as:

HSI = 8 × (ALT/AST) + BMI + 2 (if female) + 2 (if diabetic)

 Specifically, for CVD prevalence, participants with higher eGDR had a significantly lower likelihood of CVD (OR = 0.97; 95% CI = 0.96–0.98; *P* < 0.001 (Table S4 in the **Online Supplementary Document**) compared to those in the lowest eGDR quartile. Similarly, the HRs for all-cause and CVD mortality were 0.49 (95% CI = 0.35–0.70, *P* < 0.001) and 0.33 (95% CI = 0.18–0.60, *P* < 0.001), respectively (Table S5 in the **Online Supplementary Document**). Additionally, eGDR exhibited a higher C-statistic compared to other individual IR indices for both all-cause and CVD mortality outcomes (all *P* < 0.001) (Table S6 in the **Online Supplementary Document**).

## DISCUSSION

Here we examined the associations of eGDR with CVD prevalence and long-term mortality in individuals with MASLD using NHANES data. We outline three main findings. First, eGDR exhibited a distinctive L-shaped association with CVD prevalence, with risk increasing markedly at values below 5.49. Second, the relationship between eGDR and all-cause mortality was also nonlinear and L-shaped, while its association with CVD mortality followed an inverse linear gradient. Third, compared to other IR indices, including HOMA-IR and the TyG index, eGDR significantly improved mortality risk prediction, as reflected by higher C-statistics. Collectively, these results highlight eGDR as a robust marker for risk stratification and prognostication in MASLD, particularly for identifying individuals at increased cardiovascular and mortality risk.

IR is a key pathogenic driver in MASLD and its cardiovascular comorbidities [36]. In IR states, insulin’s ability to suppress hepatic gluconeogenesis and lipolysis is impaired, leading to hyperglycaemia, dyslipidaemia, and hepatic fat accumulation [37–39]. This metabolic overload promotes oxidative stress and chronic inflammation, which further disrupt insulin signalling and accelerate disease progression [40,41]. In parallel, IR diminishes insulin’s vasoprotective effects, such as nitric oxide-mediated endothelial relaxation, while promoting vasoconstriction and vascular dysfunction through endothelin and cytokine-mediated pathways [42,43]. These processes contribute to systemic endothelial injury, atherosclerosis, and increased cardiovascular risk [43,44]. Given the key role of IR in the pathogenesis of MASLD and its associated cardiovascular complications, the identification of robust markers for evaluating IR and its systemic effects is paramount. The eGDR incorporates WC, HbA1c, and hypertension status – capturing the multifaceted nature of IR. Here, HbA1c reflects chronic glycaemic exposure, hypertension indicates vascular dysfunction, and WC serves as a proxy for central adiposity and systemic inflammation. Together, these components make eGDR a more physiologically comprehensive marker of IR than glucose- or insulin-based measures alone. Earlier studies have established that eGDR is negatively associated with the risks of stroke, coronary artery disease, and all-cause mortality, among individuals with type 1 diabetes mellitus [17–19]. Furthermore, reduced eGDR levels are independently correlated with higher risks of CVD incidence in individuals without DM and mortality in the general population [20,45]. In cohorts of CKD patients without DM, increased eGDR was linked to a diminished risk of both CVD and overall mortality. Consistently, in our study, eGDR was inversely associated with the prevalence of CVD, and this relationship remained robust across most subgroups, except among individuals without obesity (*P* < 0.05). One potential explanation for the null association in non-obese individuals is that IR contributes less prominently to CVD risk in metabolically healthy or lean phenotypes. Although the observed effect sizes were modest, the consistent statistical significance across models in a large, nationally representative cohort suggests that eGDR may serve as a useful tool for risk stratification at the population level. While the clinical impact for individual patients may be limited, these associations remain epidemiologically relevant, particularly in the context of identifying high-risk subgroups for preventive cardiovascular strategies. Additionally, eGDR demonstrated a strong association with both all-cause mortality (*P* < 0.001) and CVD mortality (*P* < 0.001) in individuals with MASLD. Specifically, compared to the lowest eGDR quartile, the risk of all-cause and CVD mortality was reduced by 38% and 62%, respectively, in individuals with higher eGDR levels. Furthermore, the observed L-shaped association between eGDR and all-cause mortality suggests a steep increase in risk when eGDR falls below ~5.5, identifying a potential high-risk population. However, above this threshold, the curve flattens, indicating a saturation point beyond which further gains in insulin sensitivity provide diminishing returns. This saturation effect may reflect a transition in dominant pathophysiological mechanisms, from IR to non-IR pathways such as chronic inflammation, aging, or other comorbidities. These findings support the use of eGDR for risk stratification and suggest that interventions targeting IR may be particularly impactful in individuals below the threshold. The interaction analyses revealed that the inverse association between eGDR and mortality was more pronounced among individuals under 60 years and those with diabetes. This may be driven by the heightened impact of IR in type 2 diabetes mellitus, which contributes to chronic inflammation, endothelial dysfunction, and accelerated cardiovascular disease progression. Younger individuals may be more directly affected by metabolic abnormalities, whereas in older individuals, aging-related factors and comorbidities may attenuate the predictive power of eGDR. Furthermore, compared to traditional IR indices such as HOMA-IR and TyG, eGDR showed superior performance in mortality risk prediction based on C-statistics (*P* < 0.001), supporting its role as a clinically informative and integrative risk marker.

### Strengths and limitations

This study has several key strengths. It is the first to evaluate the prognostic utility of eGDR for mortality outcomes in US adults with MASLD, highlighting its potential as a clinical tool for risk assessment and management in this population. Additionally, it leverages a large, nationally representative cohort of US adults, with the application of sampling weights enhancing the robustness and generalisability of the findings. The relatively long mean follow-up duration of 9.74 years is another key strength, as it enables a more comprehensive assessment of the long-term impact of IR on cardiovascular outcomes – a process that often unfolds over extended periods. this study is unique in simultaneously assessing the predictive value of three IR indices for long-term mortality, providing novel insights into improving individualised risk stratification.

Despite this, we must note some of the study’s limitations. First, the diagnosis of SLD relied on an FLI ≥60 and HSI >36 rather than ultrasonographic or histological evaluation. Although FLI and HSI are advantageous in identifying individuals with MASLD [26], they lack the precision required to differentiate the specific aetiologies of SLD. Second, CVD status was based on self-reported physician diagnosis obtained through NHANES interviews, which may introduce misclassification bias in the absence of adjudication. Third, while survival status and causes of mortality were determined through links to the NDI, the cross-sectional design of NHANES precludes causal inference. Fourth, although we accounted for several potential confounders, residual confounding by unmeasured variables cannot be fully dismissed. Additionally, the calculation of the eGDR relies on baseline data, limiting the ability to evaluate longitudinal changes in eGDR and their association with MASLD outcomes over time. Lastly, as NHANES represents only the US population, external validation in diverse, multicentre cohorts is required before generalising these findings to other populations.

## CONCLUSIONS

Our findings highlight the critical role of eGDR as a valuable marker for risk stratification in individuals with MASLD. They demonstrate that lower eGDR levels are significantly associated with higher CVD prevalence and increased risks of all-cause and CVD mortality. Furthermore, eGDR outperformed traditional IR markers, including HOMA-IR and TyG index, in predicting mortality outcomes, offering new insights into its clinical utility. These results highlight a need to target IR in the management of MASLD and highlight the potential of eGDR as a clinical tool to improve early risk identification and guide personalised treatment strategies for high-risk individuals.

## Additional material


Online Supplementary Document
