# Supplementary material for: Association between estimated glucose disposal rate and cardiovascular disease prevalence and mortality outcomes in metabolic dysfunction-associated steatotic liver disease: a comparative analysis of insulin resistance markers
Source: J Glob Health. 2025 Aug 4;15:04249. doi: 10.7189/jogh.15.04249 (PMC12319350; doi:10.7189/jogh.15.04249)
Supplement: Online Supplementary Document [file jogh-15-04249-s001.pdf]

Supplement to: Chen X, Du L, Peng J. Association between estimated glucose disposal rate and cardiovascular disease prevalence and mortality outcomes in metabolic dysfunction-associated steatotic liver disease: a comparative analysis of insulin resistance markers. J Glob Health. 2025;15:04249.

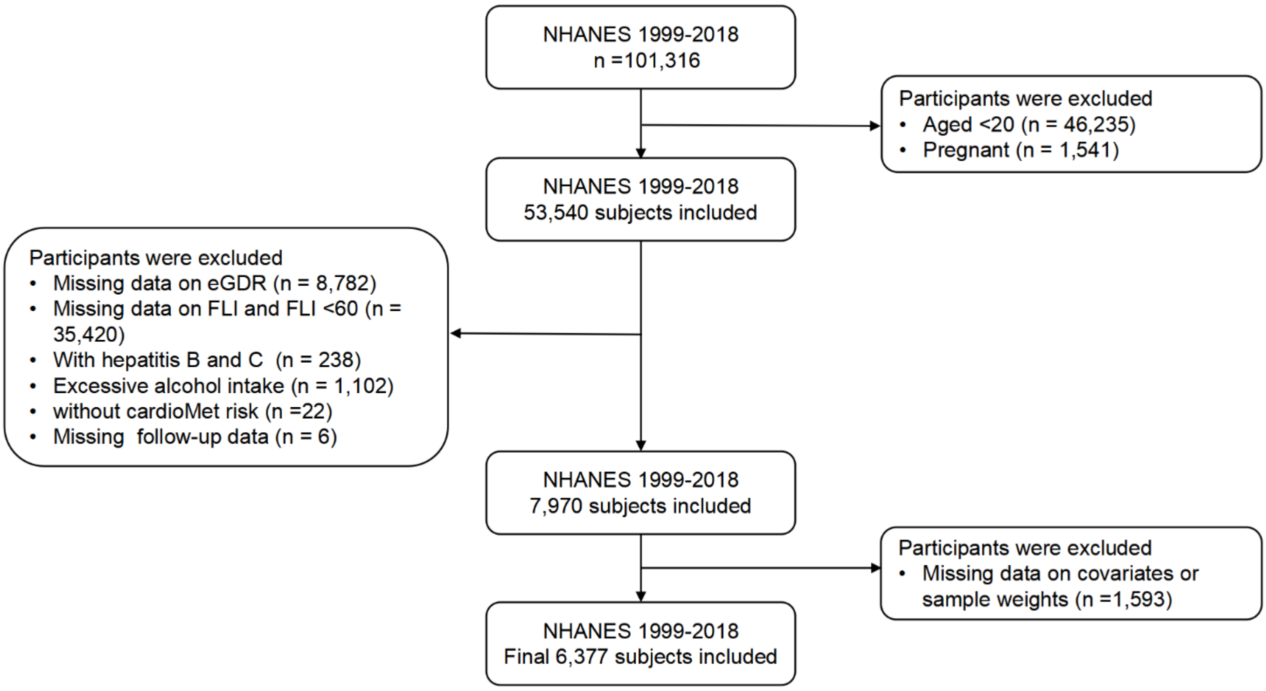

Figure S1. Flowchart of the study participants.

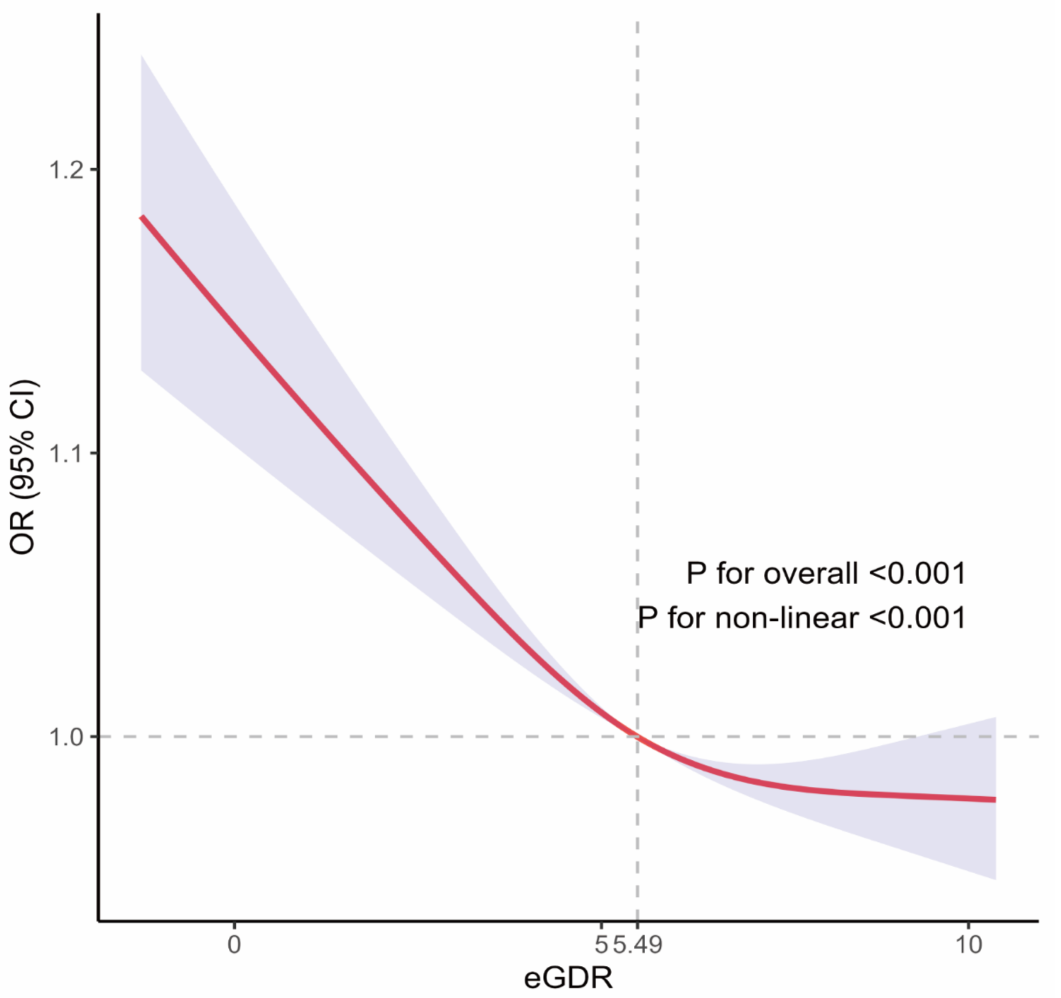

Figure S2. Restricted cubic spline curve for the association between eGDR and prevalence of CVD among individuals with MASLD. Red lines represent references for ORs, and blue areas represent 95% CIs. The model was adjusted for age, gender, race, family income poverty ratio, education level, marital status, smoking status, drinking status, energy intake, TC, ALT, AST, and eGFR. OR – odds ratio, eGFR – estimated glomerular filtration rate.

Table S1. Threshold effect analysis of eGDR on the prevalence of CVD among individuals with MASLD.

CVD	**OR (95% CI)**	***p*-value**
**Two-piecewise logistic model**		
Inflection point	5.49	
eGDR < 5.49	0.98 (0.97, 0.99)	<0.001
eGDR ≥ 5.49	1.00 (0.99, 1.00)	0.143

OR – odds Ratio, CI – confidence interval, CVD – cardiovascular disease.

Age, gender, race, family income poverty ratio, education level, marital status, smoking status, drinking status, energy intake, TC, ALT, AST, and eGFR were adjusted.

Table S2. Threshold effect analysis of eGDR on all-cause mortality among individuals with MASLD.

**All-cause mortality**	**HR (95% CI)**	***p*-value**
**Two-piecewise Cox model**		
Inflection point	5.50	
eGDR < 5.50	0.82 (0.75, 0.89)	<0.001
eGDR ≥5.50	1.02 (0.95, 1.09)	0.650

HR – hazard Ratio, CI – confidence interval.

Age, gender, race, family income poverty ratio, education level, marital status, smoking status, drinking status, energy intake, TC, ALT, AST, eGFR, and CVD were adjusted.

Table S3. Evaluation of model discrimination and risk reclassification for all-cause and cardiovascular mortality.

Model	C-statistic (95%CI)	ΔC (95%CI)	*P*-value	NRI (95% CI)	*P*-value
**All-cause mortality**					
eGDR	0.646 (0.628, 0.663)	Ref	Ref	-	-
TyG index	0.551 (0.531, 0.571)	-0.0938 (-0.1184, -0.0693)	<0.001	-	-
HOMA_IR	0.508 (0.487, 0.528)	-0.1378 (-0.1598, -0.1158)	<0.001	-	-
Basic Model	0.836 (0.823, 0.848)	Ref	Ref	Ref	
Basic Model + eGDR	0.839 (0.827, 0.851)	0.0036 (0.0014, 0.0059)	0.001	0.170 (0.108, 0.231)	<0.001
**CVD mortality**					
eGDR	0.670 (0.641, 0.699)	Ref	Ref	-	-
TyG index	0.548 (0.513, 0.584)	-0.1208 (-0.1646, -0.0769)	<0.001	-	-
HOMA_IR	0.512 (0.474, 0.549)	-0.1578 (-0.1967, -0.1189)	<0.001	-	-
Basic Model	0.860 (0.839-0.881)	Ref	Ref	Ref	
Basic Model + eGDR	0.865 (0.846-0.885)	0.0053 (0.0000, 0.0106)	0.050	0.292 (0.192, 0.386)	<0.001

NRI –net reclassification improvement, CI – confidence interval, CVD – cardiovascular disease, eGDR – estimated glucose disposal rate, TyG index – triglyceride-glucose index, HOMA_IR – homeostasis model assessment of insulin resistance, ref – reference.

Basic Model: Age, gender, race, family income poverty ratio, education level, marital status, smoking status, drinking status, energy intake, TC, ALT, AST, eGFR, and CVD were adjusted.

Table S4. Associations between eGDR and prevalence of CVD among individuals with MASLD defined by HSI.

	**Model 1**		**Model 2**		**Model 3**	
**MetS**	**OR (95% CI)**	***p*-value**	**OR (95% CI)**	***p*-value**	**OR (95% CI)**	***p*-value**
**eGDR Continuous**						
Per SD increase	0.94 (0.93, 0.94)	<0.001	0.97 (0.96, 0.98)	<0.001	0.97 (0.96, 0.98)	<0.001
**eGDR Quartiles**						
Q1	Ref		Ref		Ref	
Q2	0.92 (0.89, 0.94)	<0.001	0.93 (0.91, 0.96)	<0.001	0.94 (0.92, 0.97)	<0.001
Q3	0.86 (0.84, 0.88)	<0.001	0.91 (0.89, 0.93)	<0.001	0.92 (0.90, 0.94)	<0.001
Q4	0.84 (0.82, 0.86)	<0.001	0.92 (0.90, 0.94)	<0.001	0.93 (0.90, 0.95)	<0.001
P for trend		<0.001		<0.001		<0.001

CI – confidence interval, CVD – cardiovascular disease, eGDR – estimated glucose disposal rate, OR – odds ratio, Q – quartile, ref – reference, SD – standard deviation

*Model 1: no covariates were adjusted. Model 2: adjusted for age, gender, race, and family income poverty ratio. Model 3: adjusted for age, gender, race, family income poverty ratio, education level, marital status, smoking status, drinking status, energy intake, TC, ALT, AST, and eGFR.

Table S5. Associations between eGDR and risk of all-cause and CVD mortality among individuals with MASLD defined by HSI.

	Model 1		Model 2		Model 3	
	**HR (95% CI)**	***p*-value**	**HR (95% CI)**	***p*-value**	**HR (95% CI)**	***p*-value**
**All-cause mortality**						
eGDR continuous						
Per SD increase	0.51 (0.47, 0.56)	<0.001	0.72 (0.64, 0.82)	<0.001	0.74 (0.65, 0.83)	<0.001
eGDR Quartiles						
Q1	Ref		Ref		Ref	
Q2	0.53 (0.44, 0.65)	<0.001	0.63 (0.51, 0.77)	<0.001	0.66 (0.53, 0.82)	<0.001
Q3	0.32 (0.26, 0.41)	<0.001	0.66 (0.52, 0.84)	<0.001	0.70 (0.55, 0.90)	0.005
Q4	0.13 (0.10 0.18)	<0.001	0.47 (0.33, 0.66)	<0.001	0.49 (0.35, 0.70)	<0.001
P for trend		<0.001		<0.001		<0.001
**CVD mortality**						
eGDR continuous						

Per SD increase	0.43 (0.38, 0.48)	<0.001	0.61 (0.51, 0.72)	<0.001	0.63 (0.53, 0.75)	<0.001
eGDR Quartiles						
Q1	Ref		Ref		Ref	
Q2	0.44 (0.32, 0.60)	<0.001	0.55 (0.39, 0.77)	<0.001	0.59 (0.42, 0.82)	0.002
Q3	0.19 (0.12, 0.30)	<0.001	0.42 (0.26, 0.68)	<0.001	0.46 (0.28, 0.74)	0.001
Q4	0.07 (0.04, 0.12)	<0.001	0.30 (0.17, 0.55)	<0.001	0.33 (0.18, 0.60)	<0.001
P for trend		<0.001		<0.001		<0.001

CI – confidence interval, CVD – cardiovascular disease, eGDR – estimated glucose disposal rate, HR – hazard ratio, Q – quartile, ref – reference, SD – standard deviation

*Model 1: no covariates were adjusted. Model 2: adjusted for age, gender, race, and family income poverty ratio. Model 3: adjusted for age, gender, race, family income poverty ratio, education level, marital status, smoking status, drinking status, energy intake, TC, ALT, AST, eGFR, and CVD.

Table S6. Evaluation of model discrimination and risk reclassification for all-cause and cardiovascular mortality among individuals with MASLD defined by HSI.

Model	C-statistic (95%CI)	*P*-value	NRI (95% CI)	*P*-value
**All-cause mortality**				
eGDR	0.700 (0.682, 0.717)	Ref	-	-
TyG index	0.574 (0.553. 0.595)	<0.001	-	-
HOMA_IR	0.554 (0.532. 0.576)	<0.001	-	-

Basic Model	0.849 (0.836, 0.862)	Ref	Ref	
Basic Model + eGDR	0.853 (0.841, 0.866)	0.003	0.345 (0.281, 0.409)	<0.001
**CVD mortality**				
eGDR	0.723 (0.695, 0.752)	Ref	-	-
TyG index	0.576 (0.540, 0.612)	<0.001	-	-
HOMA_IR	0.558 (0.519, 0.597)	<0.001	-	-
Basic Model	0.871 (0.849-0.894)	Ref	Ref	
Basic Model + eGDR	0.878 (0.857-0.899)	0.033	0.471 (0.365, 0.570)	<0.001

CI – confidence interval, CVD – cardiovascular disease, eGDR – estimated glucose disposal rate, TyG index – triglyceride-glucose index, HOMA_IR – homeostasis model assessment of insulin resistance, ref – reference.

Basic Model: Age, gender, race, family income poverty ratio, education level, marital status, smoking status, drinking status, energy intake, TC, ALT, AST, eGFR, and CVD were adjusted.

Table S7. Outline of JoGH's Guidelines for Reporting Analyses of Big Data Repositories Open to the Public (GRABDROP) items*

1. Please list all papers published by each co-author in previous three years that were based on secondary analysis of a big data repository	The following studies based on NHANES data were conducted by the authors:<br>Chen X. Association of estimated glucose disposal rate with metabolic syndrome prevalence and mortality risks: a population-based study. Cardiovasc Diabetol. 2025 Jan 22;24(1):38.<br>Chen X. Neutrophil-lymphocyte ratio and systemic immune-inflammation index as predictors of cardiovascular risk and mortality in prediabetes and diabetes: a population-based study. Inflammopharmacology. 2024 Oct;32(5):3213-3227.<br>Peng J, Du LL, Ma QL. Serum glycolipids mediate the relationship of urinary bisphenols with NAFLD: analysis of a population-based, cross-sectional study. Environ Health. 2023 Jan 2;21(1):124.
2. Please explain the key elements of your study design and the use of the available datasets that make your study an original scientific contribution	This study used combined cross-sectional and longitudinal NHANES data (1999–2018) to assess the relationship between estimated glucose disposal rate (eGDR) and both cardiovascular and mortality outcomes in patients with MASLD. To our knowledge, no prior studies have examined eGDR's predictive value for CVD and mortality specifically in MASLD populations. The statistical rigor (use of survey-weighting, Cox models, and Bonferroni correction) and comparison with traditional IR markers further enhance its originality and scientific value.
3. Please list all publications that addressed similar research questions in the same dataset and indicate where you cited them in your paper	While previous studies have evaluated the association between eGDR and MASLD ("Association of estimated glucose disposal rate with risk of future metabolic dysfunction-associated steatotic liver disease and other chronic liver diseases", and "Association between estimated glucose disposal rate and metabolic dysfunction-associated steatotic liver disease and dyslipidemia in US adults"), these studies did not evaluate cardiovascular outcomes or mortality. These publications were not cited as they were published in July 2025, after

	submission of our manuscript.
4. Please explain how you addressed multiple testing through an appropriately rigorous statistical threshold and indicate this in the methods section	Bonferroni correction was applied to control for multiple testing. With three primary comparisons, the adjusted significance threshold was P = 0.017 (0.05/3), as clearly stated in the Methods section.
5. Please declare to what extent have AI chatbots been used in developing your paper and to which parts of the paper did they contribute	No AI chatbots (ChatGPT or similar) were used during the design, analysis, writing, or revision of this paper.

Adapted from: Rudan I, Song P, Adeloye D, Campbell H. Journal of Global Health's Guidelines for Reporting Analyses of Big Data Repositories Open to the Public (GRABDROP): preventing 'paper mills', duplicate publications, misuse of statistical inference, and inappropriate use of artificial intelligence. J Glob Health. 2025 Jul 1;15:01004. doi: 10.7189/jogh.15.01004. PMID: 40587200; PMCID: PMC12208284.
